# Chunking as the result of an efficiency computation trade-off

**DOI:** 10.1038/ncomms12176

**Published:** 2016-07-11

**Authors:** Pavan Ramkumar, Daniel E. Acuna, Max Berniker, Scott T. Grafton, Robert S. Turner, Konrad P. Kording

**Affiliations:** 1 Department of Physical Medicine and Rehabilitation, Northwestern University, Chicago, Illinois 60611, USA; 2 Rehabilitation Institute of Chicago, Chicago, Illinois 60611, USA; 3 School of Information Studies, Syracuse University, Syracuse, New York 13244, USA; 4 Department of Mechanical and Industrial Engineering, University of Illinois, Chicago, Illinois 60607, USA; 5 Department of Psychological and Brain Sciences, University of California, Santa Barbara, California 93106, USA; 6 Department of Neurobiology and Systems Neurosciences Institute, University of Pittsburgh, Pittsburgh, Pennsylvania 15213, USA

## Abstract

How to move efficiently is an optimal control problem, whose computational complexity grows exponentially with the horizon of the planned trajectory. Breaking a compound movement into a series of chunks, each planned over a shorter horizon can thus reduce the overall computational complexity and associated costs while limiting the achievable efficiency. This trade-off suggests a cost-effective learning strategy: to learn new movements we should start with many short chunks (to limit the cost of computation). As practice reduces the impediments to more complex computation, the chunking structure should evolve to allow progressively more efficient movements (to maximize efficiency). Here we show that monkeys learning a reaching sequence over an extended period of time adopt this strategy by performing movements that can be described as locally optimal trajectories. Chunking can thus be understood as a cost-effective strategy for producing and learning efficient movements.

## Abstract (web summary)

Complex motions can be achieved by chunking together simple movements at the cost of producing smooth, efficient trajectories. Here the authors apply a new algorithm to monkeys learning complex motor sequences and show that optimization initially occurs within small chunks that are later combined.

## Graphical abstract

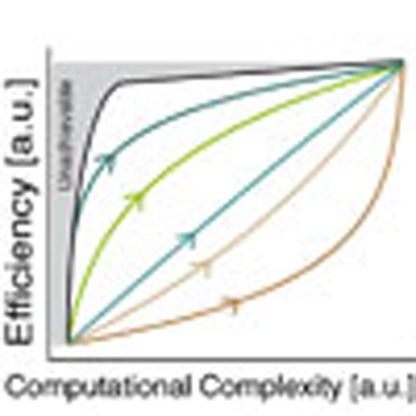

Compound movements, such as drinking a cup of tea, are typically produced by threading together a sequence of simple, elemental movements—for example, reaching out, grasping the cup, raising it to the lips, tilting it appropriately and drinking from it. Such actions tend to have distinguishable components or chunks^1^,^2^,^3^,^4^,^5^. When we first encounter a new sequence, each elemental movement is executed as an isolated chunk, that is, a continuous movement between two halts of the end effector. With learning, individual elements become faster and smoother, that is, more efficient. In addition, there is a progressive process where several contiguous elements can be combined into chunks with the result that the overall sequence is executed more efficiently and with fewer chunks^4^,^6^,^7^,^8^. The seminal chunking theory of learning^9^ describes the phenomenological signatures of movement speed up with learning (but see ref. 10), yet the causes of chunking have remained elusive. We approach the problem of why chunking occurs by considering the goals of the motor system (that is, the functions it has evolved to optimize) and ask whether chunking might be a strategy to achieve them.

In the discrete sequence production literature, chunks are interpreted as the outcome of memory processes that address the costs associated with storing and recalling long sequences from memory^11^. For instance, to remember a sequence of 10 numbers (for example, a phone number) we can combine them in a series of chunks, each three or four digits long (for example, 3–3–4). This way, instead of remembering 10 individual numbers, it is sufficient to remember three chunks. Presumably this strategy is how the brain best achieves the goal of remembering the sequence accurately while balancing the competing cost of memorizing long sequences. The vast majority of early chunking studies are built from working memory tasks and do not explicitly address the costs associated with planning or optimally executing a sequence of physical movements.

Some studies have considered movement sequences as being represented either cognitively (spatial chunks), or as synergies between muscles, joint angles and forces (motor chunks)^12^,^13^,^14^,^15^. For example, explicit instruction or knowledge experiments produce spatial chunks and implicit learning produces motor chunks^15^. Bimanual transfer experiments that discriminate between spatial and motor chunks^12^,^16^,^17^ have suggested that spatial chunks are formed earlier than motor chunks. According to a prominent theoretical synthesis^12^,^15^, control of movement sequences shifts from a general-purpose cognitive system to a specialized motor system. In this view, the motor system deals with execution while the cognitive system is freed up to attend to other tasks. Despite these studies, the literature has not considered movement sequence production from the standpoint of optimal control.

Optimal control solves the problem of determining control policies that maximize some value function. When applied to motor control, this commonly refers to the problem of computing the dynamics of joint angles and muscles that maximize efficiency (resulting in smooth trajectories and lower energetic demands of execution). To obtain smooth or efficient trajectories, a higher-order derivative of position such as the squared jerk is minimized over the course of the movement^18^,^19^. An important facet of such problems is that they are solved by dynamic programming and become exponentially harder to solve as the planning horizon of movements becomes longer^19^,^20^,^21^. Thus, the computational complexity of one extended sequence of movements as a whole is greater than the total computational complexity of shorter portions of the same sequence planned independently and then concatenated.

From these considerations, it follows that long sequences of movements can be composed of a series of computationally simpler shorter sequences, which we operationally define as chunks. However, such concatenation will in general prevent performance from being maximally efficient; the concatenation of optimal chunks is not equivalent to the optimal solution for the entire sequence. This suggests an inherent trade-off for the motor system between efficient but computationally complex long movements and computationally simple but inefficient movements comprising chunks. Thus, the trade-offs between efficiency and computational complexity must influence chunking on any given trial such that some upper bound of computational complexity and some lower bound of sufficient efficiency together determine chunk structures.

At first glance, this trade-off between efficiency and computation may appear to imply that the chunk structure, once optimized for the trade-off, should stay the same over the course of learning. However, animals clearly have the ability to form habits^22^. In the operant conditioning literature, habitual movements are defined as stimulus–response behaviours, that is, automatic actions or sequences of actions performed in response to stimuli that are insensitive to the action outcome^22^,^23^. Arguably, this automaticity of habitual movements reduces the effective cost of computation of a sequence that would have been more computationally expensive *de novo*. Moreover, there are algorithmic reasons why computation can become less costly with repeated performance. For example, algorithms can simplify the optimization process by caching partial solutions^24^, estimating cost-to-go functions^25^, or approximating the optimal policy^26^. Furthermore, the energetic benefit of an efficient trajectory is likely to offset the relative cost of added computational complexity for movements that the animal must produce several times. For all these factors, which are hard to measure experimentally, it stands to reason that the effective cost of computing more complex trajectories decreases over the course of learning. Thus, practice should enable solutions that require longer planning horizons and that result in longer chunks. We may thus intuitively expect that the trade-off between a fixed efficiency goal and a decreasing effective cost of computation gives rise to progressively longer chunks.

In this study, we tested whether monkeys produce cost-effective movement sequences using kinematic data from monkeys repeating the same sequence of centre–out reaches many times. We found that movement efficiency was optimized initially within chunks, and then gradually by producing movements of longer chunks, suggesting that monkeys are cost-effective learners. We suggest that the optimal motor control problem can be reframed as a divide-and-conquer strategy: stringing together the correct set of elemental movements into chunks and locally optimizing trajectories within the boundaries of a chunk. This reframing has broad implications for how the motor system learns to execute movement sequences efficiently.

## Results

### A movement sequence task to characterize chunking behaviour

To characterize movement-chunking behaviour in animals, we recorded arm movements from monkeys performing a sequence of centre–out reaches. Although movement sequence production has primarily been studied in human finger movement tasks, finger movements generalize less well across species. Here we intend to develop tasks to test alternative models of chunking across species so that physiological mechanisms can be studied using invasive recording techniques not available to humans. We believe that monkeys are a good model of human chunking behaviour because we are primarily interested in the control aspects of the motor system that results in chunking.

**Figure 1:**
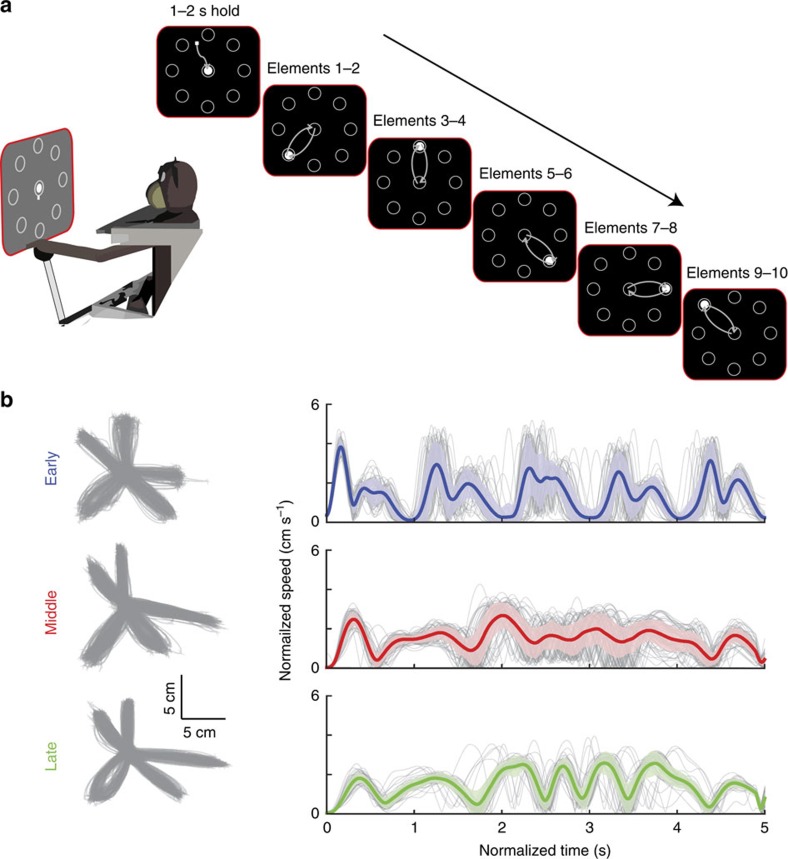
Movements become more regular with learning. (**a**) Reaching task. Monkeys move a cursor through 5 out-and-back reaches (10 elemental movements) between central and peripheral targets. White-filled circular cues indicate which target to capture. Each successful element is rewarded. (**b**) Hand trajectories: left, position; right, speed. Each trial is stretched to a duration of 5 s. Grey traces indicate single trials and bold coloured traces indicate mean traces. The coloured envelopes around the mean trace indicate one s.d. on either side of the mean.

Two monkeys (adults, *Macaca mulatta*) sequentially reached using a joystick to five outer targets, and returned to the centre target in between each reach (Fig. 1a; see Methods for details). Each target was visually cued as soon as the previous target was captured, rendering the task self-paced. The sequence was identical from trial to trial across days, and monkeys learned it through practice.

To understand how motor performance evolved with learning, we visualized speed profiles from the early, middle and late periods of learning (Fig. 1b, monkey E). In the beginning (for example, trials 1–100), the monkey is still following the spatial cues for each reach in the movement and the speed profiles are highly repeatable from one trial to the next (Fig. 1b, top panel). In the intermediate period (for example, trials 30,001–30,100), movements have decreased peak speeds and become smoother, but there is considerable variability from trial to trial (Fig. 1b, middle panel). Towards the end of learning (for example, trials 40,001–40,100), the movements are more similar from trial to trial. This increase in regularity is a signature of highly practiced movements^4^ and can be quantified using higher-order derivatives such as jerk.

### A model for achieving trade-offs through chunks

We developed a model to analyse how the trade-off between movement efficiency and computational complexity can explain the chunking observed in sequential reaching movements.

Let us define the computational complexity of optimal control as the *de novo* cost of computation (that is, the cost of computing a novel sequence of optimal trajectories). To be clear, both novel and learned sequences are associated with computational complexity, but we have no insight at present into the potential computational savings that may come with repeated performance. Therefore, we prefer to define computational complexity as the cost of computing the control trajectory for a novel sequence.

From a normative point of view, the computational complexity of planning a movement grows exponentially with the duration over which the movement is optimized^19^. To see why, consider that at each time step, the monkey can modify the kinematics (positions, joint angles and so on) and dynamics (forces, torques and so on) of its arm in *N* unique ways. To plan ahead for *T* time steps, the search space grows as *O*(*N*^*T*^). Thus, the entire sequence of movements is computationally expensive to plan but can be optimized for maximum efficiency. Alternatively, the sequence can be broken up into chunks and each chunk can be optimized independently. This scenario would be computationally simpler but not maximally efficient.

**Figure 2:**
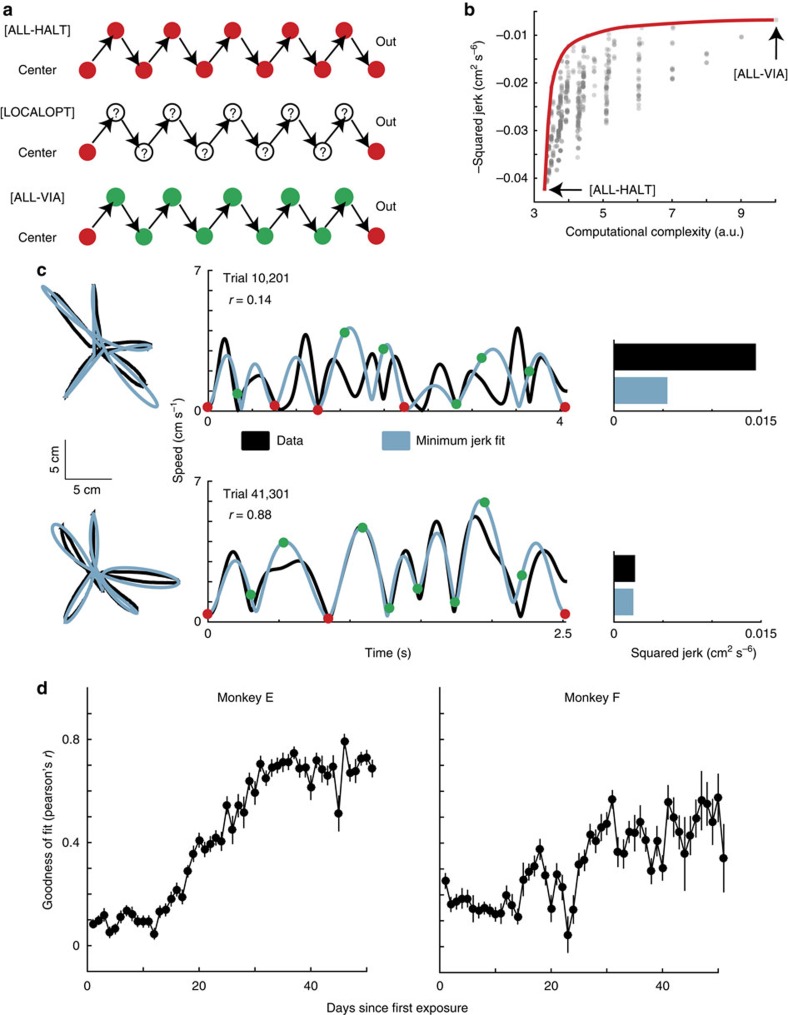
Modelling chunks as locally optimal-control trajectories. (**a**) Illustration of canonical HALT and VIA models: green, VIA points; red, HALT points. (**b**) Computing the trade-off between efficiency and the complexity of being efficient. Each grey dot represents one potential chunk structure plotted against its maximally achievable efficiency under the model, and the corresponding computational complexity. The red curve is the convex hull of these points, and represents the Pareto frontier of the efficiency–computation trade-off curve. (**c**) Kinematics (black) and minimum-jerk model (blue). Left: trajectories become more looped as monkeys optimize over longer horizons. Middle: speed traces. Initially, trajectory optimization appears to happen over several chunks. Later in learning, a smaller number of chunks reveal increasingly efficient movements. Right: the squared jerk of the kinematic data and the model suggest that the behaviour approaches the efficiency of the minimum jerk model after learning. (**d**) Goodness of fit (Pearson's correlation coefficient) between the speed profiles of the minimum-jerk model and the kinematic data (mean±2 s.e.m.'s) across days of learning.

To illustrate how this trade-off influences movement chunking in our reaching task, consider the following. In this task, we consider a single aiming movement, that is a centre–out or an out–centre movement as an elemental movement, and a combination of one or more elemental movements as a chunk. The smallest possible chunk would thus constitute a single elemental movement (for example, Fig. 1a, element 1 or 2). Thus, to minimize the computational complexity of planning an efficient movement, the arm should come to a halt at the end of every element, and each such element should be optimized independently. This scenario lies at one extreme and we refer to it as the ALL-HALT model, where each HALT point is either a centre or an outer target at which the arm comes to a halt (Fig. 2a, top panel). If however the desire for a maximally efficient movement matters most, we would expect the entire sequence to be executed as a single chunk. In this scenario, the arm would never halt and the movement would be optimized over the entire sequence. We refer to this other extreme as the ALL-VIA model, where each VIA point is a target through which the arm passes without coming to a halt (Fig. 2a, lower panel). In reality, there is a large but finite number of ways in which the entire sequence could be divided into chunks. Our model assumes that each movement sequence is executed using a grouping of chunks that lies on the continuum between these two extremes, as the animal navigates the trade-off between efficiency and computational complexity (Fig. 2a, middle panel).

Each chunk structure on this continuum is uniquely defined by specifying all potential HALT/VIA points in the trajectory, and is associated with a net computational complexity and net achievable movement efficiency. To quantify these respective computational complexities and efficiencies, we fit minimum-jerk trajectories to each trial between each pair of consecutive HALT points (chunks) and concatenated these fits across chunks (see Methods). Each such model trajectory gives the instantaneous arm position over the course of an entire movement sequence. We then quantified efficiency and computational complexity of each trajectory as follows.

In the motor control field, optimal movements are typically defined operationally as the trajectories that minimize the integrated squared jerk. Although smooth trajectories can be obtained by minimizing any higher-order derivatives of position, jerk (the third-order derivative) is typically chosen because the ratio of the peak to mean speed of model trajectories agrees well with the ratio from empirically characterized arm kinematics of human reaching movements^19^. Thus, we defined efficiency as the negative squared jerk, normalized to discount the effect of variable movement duration from trial to trial (see Methods).

To quantify the computational complexity of a movement sequence, we developed a metric that linearly sums the complexity of computing the trajectory for each chunk. Given that computational complexity of optimizing a motor control policy increases exponentially with the horizon of planning, we defined the computational complexity of a single chunk as the exponent of the number of elements, that is, the number of centre–out and out–centre reaches constituting the chunk (see Methods).

Visualizing efficiency against computational complexity for each model trajectory gives us an understanding of the trade-off (Fig. 2b; grey dots). The upper envelope of the space spanned by all potential outcomes of the model gives us an estimate of the Pareto frontier (Fig. 2b; red curve). In optimization problems that involve dual objectives, the Pareto frontier is the set of all points at which gaining ground on one objective will necessarily lose ground on another^27^. This Pareto frontier represents the trade-off curve, which gives the theoretically maximum achievable efficiency for any given complexity, and below which all real-world movements must lie.

The shape of this trade-off curve, showing diminishing returns in efficiency as a function of growing computational complexity, has two important implications. First, the efficiency gains above a certain computational complexity are marginal. This suggests that optimizing the sequence as one smooth, continuous movement may never be worth the computational effort and might explain the fundamentally discrete nature of long compound movements. Second, significant savings in cumulative computational effort might be achieved over the course of several trials, either by transitioning between chunk structures of the same computational complexity or by optimizing for efficiency within chunks. Learning to exploit this advantage inherent to the task may enable more efficient compound movements for the same amount of computation.

To test these implications of our chunking model, we analysed kinematic data from monkeys performing the centre–out sequence. First, we inferred chunk boundaries based on local minima in speed (Fig. 2c, black traces) below an adaptive threshold (see Methods). Through this method, we could parameterize each compound movement in terms of its chunk structure, which is uniquely specified by the number of elements in each chunk and the location of chunk boundaries (the HALT points). In early trials, the arm stopped at several HALT points (Fig. 2c, red dots), whereas in later trials the HALT points became VIA points (Fig. 2c, green dots). Thus, the number of chunks appeared to decrease as a function of learning.

We then modelled these kinematic data by fitting minimum-jerk trajectories between consecutive pairs of HALT points (Fig. 2c, blue traces). If the monkeys optimize for minimum jerk, we can expect the model to better explain kinematic data as movements become more efficient. Indeed, we found that the goodness of fit, quantified by the Pearson's correlation coefficient between model and data speed profiles, improved over the course of learning (Fig. 2d). This suggests that minimum jerk is a good measure of efficiency and that compound movement sequences can be modelled as locally optimal trajectories, where optimization takes place within chunks.

### Movement efficiency and computational complexity

**Figure 3:**
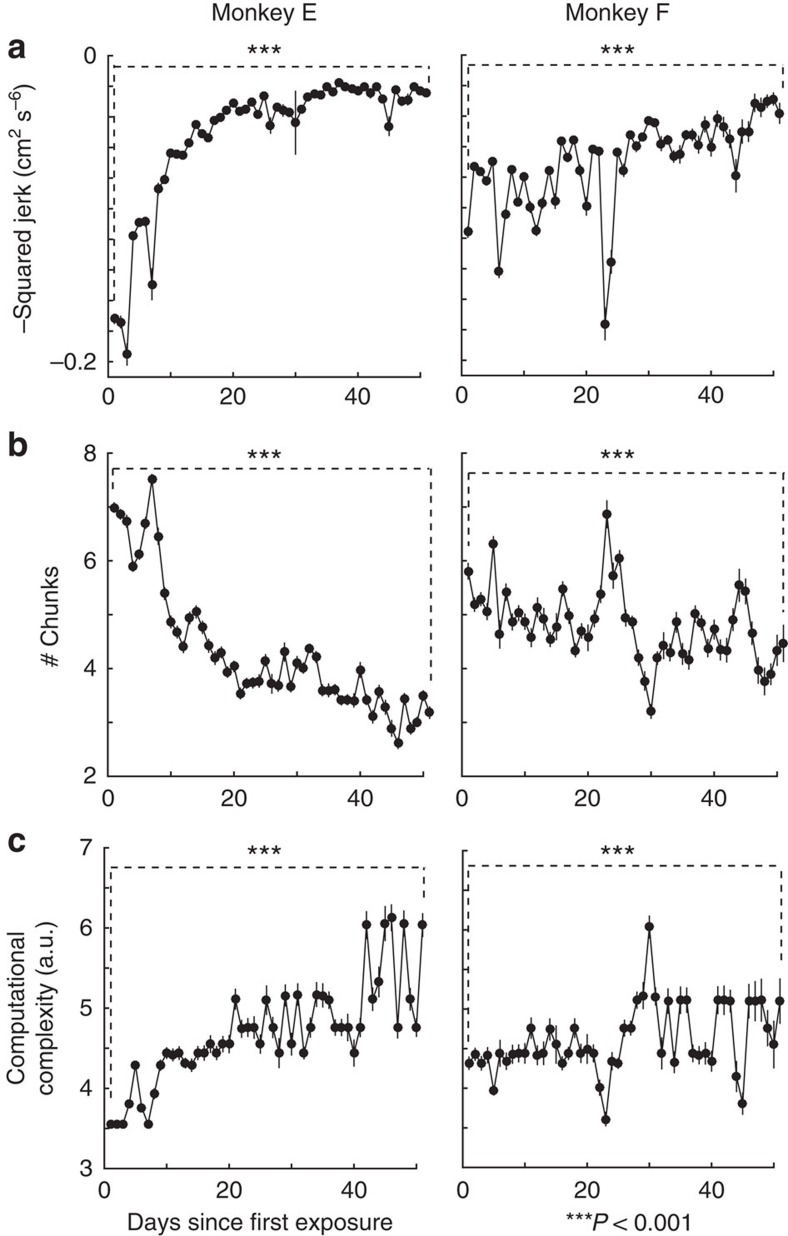
Efficiency and costs are traded off. (**a**) Over the course of practice, movement efficiency (negative normalized squared jerk; mean±2 s.e.m.'s) increases with the number of days. (**b**) The number of chunks (mean±2 s.e.m.'s) estimated by the model decreases with number of days. (**c**) Computational complexity (median±2 s.e.m.'s) of chunk structures, defined as the *de novo* cost of computation, increases with increasing chunk length due to longer planning horizons. ***P<0.001.

As suggested by our model fits to data (Fig. 2d), movements progressively resemble minimum-jerk trajectories. Independent of our model, we found that efficiency, as quantified by negative normalized squared jerk, increased by ∼50–90% over ∼50 days of performing the same sequence (Fig. 3a; unpaired two-sided *t*-test comparing trials across first and last days of practice; *P*<0.001; *n*=661 and 591 trials, respectively, for monkey E; *n*=390 and 279 trials, respectively, for monkey F). Thus, as is often observed, movement efficiency improved with learning.

Visualizing example trials (Fig. 2c) we observed that later trials had fewer chunks. Across all data, we found that the number of chunks decreased with extensive practice (Fig. 3b; unpaired two-sided *t*-test comparing trials across first and last days of practice; *P*<0.001; *n*=661 and 591 trials, respectively, for monkey E; *n*=390 and 279 trials, respectively, for monkey F). The decrease in number of chunks suggests that the length of individual chunks increases. As chunks become longer over the course of learning, movements are optimized over increasingly longer horizons.

Repeated execution of certain sequences should enable savings in the cost of computation. Thus, with practice, the motor system can select chunk structures with fewer and longer chunks. Although computational complexity is directly related to chunk length, the relationship is still non-monotonic in nature. For instance, the trajectory for a chunk structure of 8–1–1 is more computationally complex than a chunk structure of 5–5, even though the former has more chunks than the latter. Therefore, we explicitly tracked the computational complexity—which can be thought of as the cost of computing a chunk structure *de novo*—as a function of learning. We found that this metric increased with learning (Fig. 3c; unpaired two-sided *t*-test comparing trials across first and last days of learning; *P*<0.001; *n*=661 and 591 trials, respectively, for monkey E; *n*=390 and 279 trials, respectively, for monkey F). Thus, a large number of chunks early on in learning keeps the cost of computation low. As learning simplifies computational solutions, the motor system is able to optimize over progressively longer horizons, resulting in the selection of chunk structures with greater computational complexity.

The observed decrease in number of chunks could be attributed to a general increase in movement speed and reduction in reaction times, leading to detection of fewer chunk boundaries over time simply because local minima of speed profiles fall below the boundary detection threshold accidentally. This would imply that the observed chunk structures are random and do not converge towards more efficient ones. To rule out this possibility, we performed two control analyses.

First, if it were indeed true that the decrease in number of chunks was an apparent phenomenon resulting from faster movements, we would expect the number of unique chunk structures executed in a single day to remain unchanged as a function of learning. However, we found that the number of unique structures per day decreased with learning (Supplementary Fig. 1), suggesting a convergence towards chunk structures that improve efficiency of the entire sequence. Second, if chunks resulted purely from speeded-up movements, we would expect high variability in chunk structure and low correlation between consecutive trials. To test this, we computed the Hamming distance between chunk structures of consecutive trials, defined as the number of VIA to HALT and HALT to VIA point transitions. We found that even during the intermediate periods of learning with greatest variability, the average Hamming distance does not exceed 3 (Supplementary Fig. 2), even though the expected Hamming distance would be 4.5 if the chunk structures were selected at random on each trial. Together, these controls suggest a phenomenon involving the noisy evolution of chunking patterns towards increasing efficiency.

### Cost-effective learning behaviour

Optimizing movements for efficiency requires computational resources. For a given amount of computational resources, there is a limit to the possible range of movements. Reiterating, this implies the existence of a Pareto frontier that describes the computational complexity of the problem that needs to be solved for a given movement efficiency, and constitutes a constraint the motor system must contend with.

**Figure 4:**
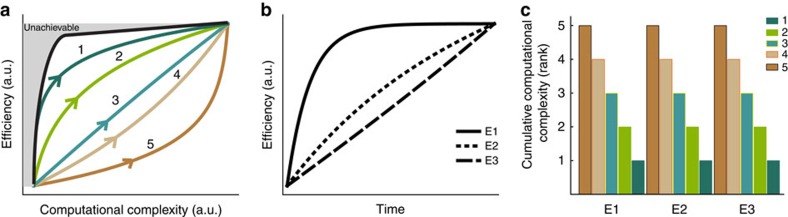
The effect of chunking on cumulative computational resources. (**a**) The black trace shows the Pareto frontier representing the maximum efficiency that can be achieved for a given complexity. Thus, the greyed-out region cannot be achieved. This represents a fundamental trade-off between efficiency and computational complexity. The coloured paths illustrate potential learning strategies, with increasing budgets from scenarios 1 through 5 for the cumulative outlay of computation. (**b**) For different rates of efficiency improvement as a function of trials (curves E1–E3), we can compute the cumulative computation of each learning strategy. (**c**) Each learning strategy is associated with a different cumulative outlay of computation over the course of learning. These outlays are ranked for the three efficiency improvement rate curves (E1–E3), with rank 1 indicating the least cumulative outlay of computation and outlays increasing from scenario 1 to 5.

There are multiple strategies (Fig. 4a, coloured traces) to transition from a naive (low efficiency and low complexity) to a practiced (high efficiency and high complexity) movement in the efficiency–computation space. A learner with limited computational resources over the course of learning would take a vertical path and only take on trajectories of greater computational complexity when even further efficiencies are required or when the effective cost of computation decreases (for example, through mechanisms associated with habit formation; Fig. 4a; scenarios 1 and 2). Alternatively, a learner may choose to devote increasing computational resources constantly throughout learning (Fig. 4a; scenario 3). Finally, deploying greater computational resources right from the outset provides maximal achievable efficiency immediately. Learners using this strategy optimize movements over long horizons at high complexity (Fig. 4a; scenarios 4 and 5). Critically, these different learning scenarios are not equivalent with respect to the cumulative computational resources devoted over the course of learning.

To quantify the cumulative outlay of computational resources associated with each strategy, we performed a simulation based on the known ways in which organisms improve efficiency over time (see Methods). Across a wide range of rates of increase in efficiency (Fig. 4b), scenario 1 ranks first in terms of minimizing the total cost of computation (Fig. 4c), even though all scenarios achieve the same efficiency (see Methods for details). Thus, a chunking-based strategy described by following the Pareto frontier in the efficiency–computation space results in the smallest total outlay of computational resources over the course of learning.

**Figure 5:**
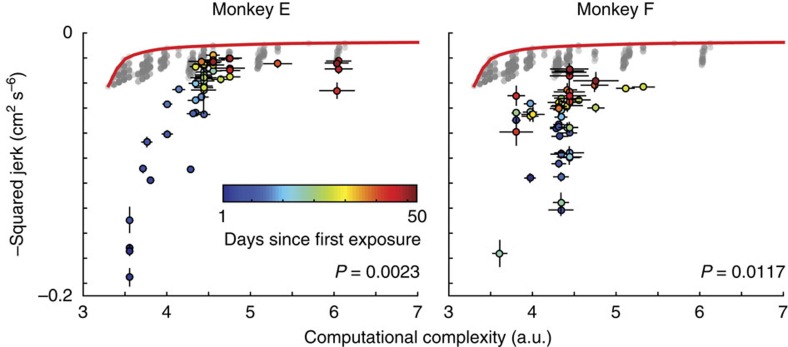
Monkeys learn to be efficient cost-effectively. They first increase their efficiency at low computational complexity and then select chunk structures with greater complexity to achieve even greater efficiencies. Coloured dots show mean efficiency against computational complexity for each day of learning, going from blue to red over the course of learning. Error bars show 2 s.e.m.'s across trials in each day. Grey dots show all possible minimum-jerk model trajectories plotted against their respective computational complexity. The red trace shows the best achievable frontier of the efficiency–computation trade-off.

To test this hypothesis, we visualized how motor performance evolved through learning along the dimensions of efficiency and computational complexity (Fig. 5, coloured dots). By comparing behavioural data against the complexity and efficiency of the minimum-jerk model trajectories (Fig. 5, grey dots) and the Pareto frontier (Fig. 5, red trace), several aspects stand out. First, the efficiencies of the model trajectories are much higher than those of actual behaviour during the first several days of learning. Thus, when the monkeys start learning, their efficiency is low (their jerk is far from the minimum) but it approaches the maximum efficiency (minimum jerk) after several days of learning.

Second, even after tens of thousands of trials, the monkeys do not reach the point where the entire sequence is executed as a single chunk. Indeed, the average computational complexity of chunk structures in a session seems to be restricted to about 50–60% of the complexity of executing the sequence as a single chunk (Fig. 5 versus Fig. 2b). This suggests that even if reductions in the cost of computation can be achieved by habit learning, they are not arbitrarily large reductions to the point that such costs do not matter. Given this and the diminishing returns of efficiency with increasing complexity, it seems that the persistence of chunking far into learning is a reasonable strategy.

Third, we find that the two monkeys took different learning paths while learning to produce efficient movement sequences. Monkey E took a path that very closely resembles the most cost-effective strategies (Fig. 4; scenarios 1 and 2) by increasing efficiency without increasing computational complexity for the first half of the sessions and then selecting chunk structures with greater computational complexity to achieve further efficiency improvements during the second half of the sessions. By contrast, monkey F took a path with no appreciable increase in computational complexity during the entire period of learning. Thus, it seems to achieve efficiency improvements for a fixed complexity. How cost-effective are these different strategies with respect to the outlay of computational resources over the course of learning?

To quantify the cost-effectiveness of these learning strategies, we used a Monte Carlo simulation. We built a null hypothesis under which monkeys aim to achieve the efficiency gains observed in the experiment with no regard for the cumulative outlay of computation throughout learning. Such a learner would show random changes in average cost from day to day. We simulated this behaviour using a random walk through the space of computational complexity from one day to the next, and a deterministic update of efficiency for each day based on data. We simulated a large number of these learning paths to represent candidates from the null distribution (see Methods). We found that the null hypothesis could be rejected at a significance level of *P*=0.0023, *n*=51 days in monkey E and *P*=0.0117, *n*=51 days in monkey F). This suggests that monkeys choose a learning strategy that is highly cost-effective.

### Movements are optimized within chunks

**Figure 6:**
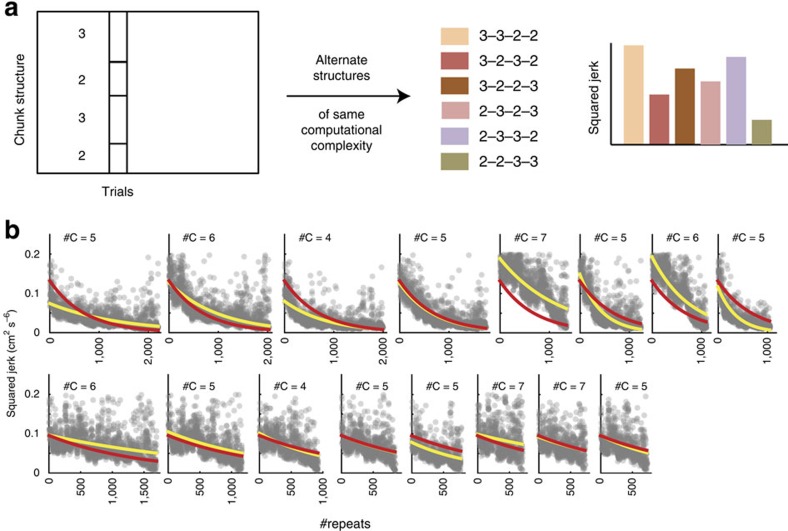
Optimization of efficiency at fixed computational complexity. (**a**) Method to identify degenerate sets of chunk structures with the same computational complexity. (**b**) Efficiency improves for fixed computational complexity with learning. Top panel: monkey E; bottom panel: monkey F. The squared jerk decreases as a function of trials for each degenerate set of chunks (the eight most commonly occurring sets are shown). The number of chunks for each degenerate set is indicated in the header of each plot. The red fit line is the fixed effect model that is identical for each set and the yellow fit line is the mixed effect model that is unique to each set.

We have shown that monkeys learn by navigating an efficiency–computation trade-off cost-effectively although they use different learning strategies (Fig. 5). Efficiency improvements can be achieved in two ways. First, the motor system could be optimizing movements within chunks. Second, by exploiting the fact that many chunk structures share the same computational complexity (Fig. 6a), the motor system could swap chunk boundaries in a manner that preferentially selects high-efficiency chunks for a given cost. To distinguish between these possibilities, we analysed whether movement efficiency improves without changing computational complexity, and whether chunk structures within a given complexity are preferentially selected for their efficiency.

First, we analysed improvements in efficiency for trials with matched computational complexity. We identified degenerate sets—which we define as the set of all chunk structures with identical complexity—as follows. Denoting a chunk structure as *M*_1_–*M*_2_–...–*M*_*k*_, where *M*_*j*_ is the number of elements in the *j*-th chunk out of *k* chunks, the complexity is identical for all possible chunk structures that only differ by a permutation of {*M*_*j*_}. That is, any two movements have the same computational complexity as long as they contain chunks with the same numbers of elements in each chunk (for example, 3–2–3–2, and 3–3–2–2 (Fig. 6a). We determined if efficiency was optimized without changing computational complexity by analysing how normalized squared jerk changes with learning for chunk structures within each degenerate set.

We found that efficiency increased over time within degenerate sets (Fig. 6b). For the eight most frequently occurring degenerate sets (among 42), comprising over 70% of all trials, we quantified the rate at which squared jerk changed with time. We used a nonlinear mixed effects model to separate out the ‘random effect' arising from the degenerate set, and the ‘fixed effect' of interest: squared jerk as a function of repeats (see Online Methods). We found a significant effect of learning on squared jerk (*z*-test; *z*=–6.1; *P*<10^–9^, *n*=12,699 trials for monkey E; *z*-test; *z*=–9.0; *P*<10^–10^, *n*=7,576 trials for monkey F). Thus, monkeys are capable of optimizing efficiency without altering the complexity of chunks.

The optimization of efficiency at fixed computational complexity does not rule out the possibility that the monkey may swap chunk boundaries to select more efficient chunks within degenerate sets, for example, 2–2–3–3 instead of 3–3–2–2. If fixed-complexity efficiency gains were indeed being achieved through this strategy, we should observe a greater prevalence of higher-efficiency chunk structures among all possible chunk structures for a majority of degenerate sets. To test this, we performed a ranking analysis as follows. For each degenerate set (described above), we estimated the frequency of prevalence of each chunk structure in the learning task. If chunks are indeed selected for their efficiency, we should expect that this frequency distribution within a degenerate set must be positively correlated with efficiency. We found no such evidence (Spearman's rank correlation across *n*=42 degenerate sets, *ρ*=–0.13 for monkey E; *ρ*=–0.20 for monkey F; *P*>0.05 for both). This suggests that the observed efficiency gains without an increase in computational complexity result from optimization of trajectories within chunks.

## Discussion

In this study, we proposed that the observed discretization of compound movements into chunks emerges from the trade-offs between different goals of the motor system: maximizing movement efficiency and minimizing computational complexity. By operationalizing the definitions of efficiency and computational complexity in a simple model, we showed that chunking is a potential strategy of the motor system to produce efficient movements while keeping computational complexity tractable. We found evidence that overall computational complexity is kept in check over the course of learning largely due to the optimization of movements within chunks. Thus, chunking is a cost-effective strategy of learning to make efficient movements.

One strategy for improving efficiency at a fixed complexity is for the animals to progressively switch to chunk structures having the same computational complexity (that is, the same number of chunks and the same chunk lengths up to a permutation) but higher efficiency (for example, 3–3–2–2 versus 2–2–3–3). However, we found no evidence that either animal did anything more sophisticated than optimizing trajectories within chunks. Although selecting chunk structures for efficiency within a degenerate set is an attractive strategy, having to search across chunk structures of equivalent complexity requires prior knowledge or internal models of efficiency, which animals may not possess. Further, re-learning a new chunk structure from one trial to the next might impose extra costs on working memory. Finally, any reduction of computational complexity gained by practicing one particular structure will probably not transfer to the new more efficient chunk. Thus, optimizing within chunks, even when better, unexplored chunk structures exist, might be a simple and effective strategy.

The model assumes that the movement time between targets is the same. We made this simplifying assumption due to the practical concerns of limiting the number of free parameters. This is a reasonable assumption for a structured laboratory task such as reaching between points, but we do not know how well this would generalize to a more complex natural movement sequence with different speed–accuracy requirements. Extending the model's capabilities to handle more natural movements is an important future direction.

Our modelling of the computational complexity is based on the underlying assumption that each unit of computation is allocated towards optimizing trajectories between chunk boundaries as opposed to planning suboptimal trajectories over longer horizons with fewer boundaries. Although these assumptions may be an oversimplification, there are no known studies quantifying the relative effort for the nervous system to optimize a trajectory with respect to executing it. Addressing these limitations by developing better metrics for computational complexity or by experimentally measuring it would be important avenues of future work.

The literature on discrete sequence learning has suggested that as behaviours become more automatic, they become cheaper to plan and execute^12^,^15^,^16^. Indeed, it is possible that the nervous system has strategies to make costs cheaper through extensive learning. For instance, parts of the optimal solution might already be stored or indexed, which, over time, would render costs of computation lower^25^. This may allow a strategy where over time longer trajectories are optimized at the same real cost as the *de novo* cost of shorter trajectories. Further, these savings in computational cost may also go hand in hand with efficiency gains in encoding long chunks into memory, which can be reorganized to make the retrieval cost of long movement chains smaller. Thus, further work is required to understand how to compare the relative difficulty of computing optimal control with those underlying attention and memory, and how this effort changes with time.

One apparently counterintuitive finding in our results is that computational complexity increases with learning. This observation goes against our everyday intuition that habitual movements feel cognitively effortless. However, here we use a restrictive definition of computational complexity: the cost of computing the control sequence for producing a desired movement trajectory. The brain has to compute a control sequence for virtually all our movements, and it is currently unresolved as to whether such control is computed by a conscious cognitive system, or whether it is computed by a motor system whose activity is inaccessible to cognitive systems. Recent work in movement chunking^15^ suggests that the computation of control for everyday movements occurs at a level below conscious awareness. Therefore, even if computational complexity increases as movements become habitual, they are likely to feel cognitively effortless, and the observed increase in complexity with learning can coexist with the effortlessness of habitual movements.

Our model suggests that efficient control trajectories can be obtained with a divide-and-conquer strategy. If each chunk is an independently optimized trajectory, then the task of computing a motor command is reduced to a set of yes or no decisions (2^*N*–1^ HALT or VIA points for *N* movement elements), and the computation of locally optimal trajectories. Thus, our model reconceptualizes motor control as having a novel decision-making component; that is, movements are not inherently discrete due to a command-generating mechanism, but rather due to decisions regarding how to structure locally efficient movements, and learn cost-effectively. This is similar to the strategy used in optimal control when choices of contact points are considered^28^. Such strategies that use hybrid optimization can allow highly efficient approximate solutions for complex problems.

One of the key challenges of modelling movement sequence learning in an optimal control setting is to be able to explain why animals do not perform the optimal trajectory in a single shot. As such, it is useful to point out that optimal control theory specifies the necessary and sufficient conditions for a desired behaviour through a cost. However, the theory as applied to biological motor control does not concern itself with the algorithms that describe how optimal control is learned. Indeed, future work in this direction will help integrate principles of optimal control theory with theories of motor learning and may potentially provide normative models of how chunk structures must evolve with learning.

The significant trial-to-trial variability of chunk structures that persists well after extended practice deserves further examination, particularly because it diverges from the typical notion of chunks in the literature as relatively robust^12^,^15^. This observation suggests that although chunks may develop with learning, they need not be used in each trial and new chunk structures can be developed even after extended practice. Therefore, in future work it would be important to develop a better understanding of the contexts in which the control for a movement sequence is computed online in each trial, and the contexts in which the control is stored and reproduced.

Chunks play a key role in the ability of the nervous system to efficiently learn, store and recall motor procedures such as walking, speaking or playing musical instruments^22^. Moreover, impairments in initiation and completion of sequential movements are a key factor of several neurological disorders involving the basal ganglia such as Parkinson's or Huntington's disease^23^,^29^,^30^,^31^. Chunking is also widely observed while learning new compensatory movements in patients recovering from stroke^5^,^32^. One potential interpretation of these disorders under our framework is that the mechanism underlying chunking is affected by the disorder, and local optimization needs to be re-learned as a result. In this view, the high-level goal of rehabilitation may be recast as a set of interventions that effectively help to re-learn local optimization. Therefore, understanding the relationship between chunking and movement disorders is important for movement rehabilitation.

We have proposed an explanation for the discrete nature of movements based on the goals of the motor system. Although earlier studies have considered how the goals of the motor system, including effort and efficiency, might influence motor adaptation and learning^33^,^34^,^35^, the interplay between computational complexity and efficiency needs further examination. The neural substrates and mechanisms subserving the motor system's goals are only beginning to be understood. Recent neurophysiology studies have shown that the basal ganglia are critical for learning to perform sequences of actions^32^,^36^,^37^,^38^,^39^,^40^. Since basal ganglia and inferior frontal gyrus circuits have been implicated in sequencing cognitive actions, chunking of motor sequences^1^,^33^,^36^,^37^,^38^,^39^,^40^,^41^,^42^,^43^,^44^ and regulating the efficiency of movement^45^,^46^,^47^, they may have a potential role in computing the trade-off between computational cost and efficiency needs. The exact way in which they do so is unknown, however. Discovering their role in shaping the motor system's goals is a promising direction for future neurophysiological experiments.

## Methods

### Experiments

All animal procedures were approved by the Institutional Animal Care and Use Committee and complied with the Public Health Service Policy on the humane care and use of laboratory animals. Two female non-human primates (*Macaca mulatta*), aged 6.5 and 5 years, weighing 6.5 and 5.7 kg, respectively, executed multiple trials of the same five-target sequence of centre–out reaches using a joystick whose position was mapped to an on-screen cursor. Starting from the centre, the monkeys had to reach to an outer target at a radial distance of ∼7 cm. After capturing the outer target with the cursor, they had to return to the centre target, after which a drop of food reward was delivered and the next outer target was shown (Fig. 1a). Although it might have been ideal to reward the monkeys at the end of each sequence just once, we found that in practice they were more willing to work if rewarded at the end of each out–centre return reach. This centre–out–centre pattern was repeated five times through the entire sequence. Each centre and outer target was visually cued as soon as the previous target was captured. The sequence was executed in a single trial and the inter-trial interval varied between 1 and 2 s. Both monkeys performed their respective sequences over multiple days (monkey E: 51 days, 41,865 trials; monkey F: 51 days, 28,951 trials). We measured the position of the arm (joystick) at 1,000 Hz. We discarded trials in which monkeys did not complete the entire sequence. We also discarded outliers of squared jerk, larger than 0.2 cm^2^ s^−6^ (504 trials in monkey E and 260 trials in monkey F), which was very close to the 95-percentile threshold in both monkeys.

### Detecting chunk boundaries

We detected chunk boundaries (that is, HALT points) for each trial based on local minima of speed trajectories that dropped below an adaptive threshold (5% of the peak speed of that trial). If more than one local minimum was detected in between two local maxima that exceeded a threshold (25% of the peak speed of that trial), we only retained the lowest minimum among these local minima.

To compute the speed trajectory itself, we smoothed *x* and *y* positions using a finite-impulse response low-pass filter having a 100-ms support, and then took the finite-difference derivate to calculate *x* and *y* velocities. The speed was computed as the square root of the sum of squares of *x* and *y* velocities.

### Quantifying efficiency and computational complexity

To quantify movement efficiency, we calculated sum of the squared jerk for each trial. Monkeys completed the sequences in shorter durations with learning, and the expected increase in sum squared jerk from faster movements scales as a fifth power of increase in duration. Since we were interested in decreasing trends in jerk that could not be predicted by duration changes alone, we stretched the duration of every trial to be exactly 5 s. We then computed jerk as the sum of squares of the third derivatives of *x* and *y* positions. Before taking each derivative, we smoothed the kinematic estimates (position, velocity and acceleration) using a finite-impulse response low-pass filter having a 100-ms support.

To quantify computational complexity, we exploited the idea that the complexity of the search space grows exponentially with the planning horizon^19^. We defined the computational complexity of optimizing an *n*-element chunk to be proportional to exp(*n*). Thus, for a sequence comprising *K* chunks (as inferred by our chunk boundary detection method), each of length *n*_1_, *n*_2_, …, *n*_*k*_, we defined the complexity as 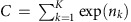. To improve the interpretability of this metric, we scaled complexity to units of chunk length and used log(*C*) as a measure of complexity.

### Minimum-jerk modelling

We used a minimum-jerk trajectory framework to formally test the hypothesis that movements are optimized within chunks. Squared jerk is a measure of optimality in the context of movement efficiency, and optimal control trajectories between two points are defined as those that minimize the jerk^20^,^21^. It can be shown using the calculus of variations that the integral of squared jerk over the duration of a movement can be minimized by a polynomial function of duration whose sixth derivative is zero^18^,^19^. Thus, in the simplest case of a straight reach between two points, minimum-jerk optimization is a simple constrained-optimization problem in which the movement trajectory is modelled as a fifth-order polynomial of the duration^18^,^19^. We used a more advanced variant of this method^21^ that maximizes the smoothness of a trajectory given a set of initial conditions—the start and end positions and velocities, as well as the set of all points that must be traversed at specified times.

Given our hypothesis that the desire for efficiency influences chunk structure in movement sequences, we operationally defined each chunk as an optimal-control trajectory that minimized squared jerk. We applied this framework to compute a model trajectory for each possible chunk structure. We allowed the transition between each element (in our case, from a centre–out to an out–centre reach, or vice versa) to be either a VIA point or a HALT point. For our 10-element sequence, this resulted in 9 binary parameters and 2^9^=512 possible chunk structures.

For initial conditions, we assumed zero velocities at the beginning and at the end of each sequence. We also assumed that the hand started and finished at a centre target at (0, 0), and traversed through outer targets at a radial distance of 7 cm from the centre. We further assumed that each centre–out or out–centre reach was executed in exactly 0.5 s; thus, the entire reach sequence would last 5 s. With these assumptions, we precomputed the locally optimal hand trajectory for all 512 possible chunk structures. Each of these trajectories is associated with a computational cost and efficiency. By plotting these against each other and computing the convex hull, we computed the Pareto frontier representing the trade-off between computational cost and efficiency.

To fit minimum-jerk models to data, for each trial, we inferred chunk boundaries from local minima of speed profiles (see above). Given these chunk boundaries, we fit a minimum-jerk trajectory for each chunk. We then assessed the goodness of fit of the model using the Pearson's correlation coefficient between speed trajectories of the model and the data.

### Calculating the cost-effectiveness of learning paths

We contend that based on the known ways in which organisms improve efficiency over time, moving along the Pareto frontier in the efficiency–computation space produces the least cumulative cost of computation over the course of learning. To demonstrate this, we performed a simulation.

First, we parameterized the efficiency improvements with time. It is widely known that organisms improve efficiency exponential over time. Let us assume that this exponential improvement starts from zero efficiency and ends at unity efficiency from time zero to time unity following the form:


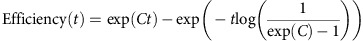


where 0<*C*<½ produces exponential improvements. Further, let us assume that the efficiency–computation space can be traversed using a similar exponential form, where complexity depends on efficiency as follows:


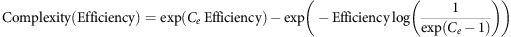


where *C*_*e*_ defines whether the curve would follow along the Pareto frontier (0<*C*_*e*_<½) or away from it (*C*_*e*_>½). We can define the cumulative complexity over time as


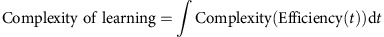


Assuming an exponential improvement in efficiency over time and using numerical integration, the cumulative complexity of learning is minimized when the efficiency–computation space is traversed close to the Pareto frontier (Fig. 4).

### Efficiency changes for fixed computational complexity

To examine how efficiency improves when computational complexity does not change, we defined sets of degenerate chunk structures that have the same complexity (Fig. 6a). We selected the eight most frequently observed sets that covered >70% of all analysed trials, for further analysis. For these eight sets, we fit a hierarchical exponential mixed effects model with the fixed effect capturing squared jerk as a function of repeats and the random effect capturing the differences across the eight degenerate sets. We then tested the fixed effect for statistical significance using a *z*-test on the exponential parameter *b* of the exponential model: 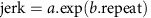.

### Quantifying the cost-effectiveness of learning

To statistically test whether monkeys followed a cost-effective learning strategy as opposed to a random strategy, we performed Monte Carlo simulation. Given the efficiency of a naive monkey (efficiency on day 1), the number of days of learning and an efficiency goal (of –0.01 cm^2^ s^−6^), we simulated a random walker to transition through the space of complexity. At each day (step), the state of the random walker could transition from one point in the complexity space to another, based on a transition probability distribution that was estimated by fitting a Gaussian to the empirical distribution of change in complexity from one day to the next. By simulating 5,000 random walkers, we built a distribution of learning paths for the null hypothesis that the monkeys only care about efficiency without concern for minimizing computational complexity over the course of learning. To make the null distribution generalizable across the data from both monkeys, we computed the average complexity per day for each path in the distribution. By comparing true behaviour against this null distribution, and assigning a percentile score for the actual learning paths adopted by each animal, it was possible to quantify the cost-effectiveness of their respective learning strategies.

### Data availability

All relevant data will be provided on request.

## Additional information

**How to cite this article:** Ramkumar, P. *et al.* Chunking as the result of an efficiency computation trade-off. *Nat. Commun.* 7:12176 doi: 10.1038/ncomms12176 (2016).

## Supplementary Material

Supplementary Information Supplementary Figures 1 and 2

## References

[b1] Morasso P., Mussa-Ivaldi F. & Ruggiero C. How a discontinuous mechanism can produce continuous patterns in trajectory formation and handwriting. Acta Psychol. 54, 83–98 (1983).

[b2] Milner T. E. A model for the generation of movements requiring endpoint precision. Neuroscience 49, 365–374 (1992). 1436478 10.1016/0306-4522(92)90113-g

[b3] Vallbo A. B. & Wessberg J. Organization of motor output in slow finger movements in man. J. Physiol. 469, 673–691 (1993). 8271223 10.1113/jphysiol.1993.sp019837 PMC1143894

[b4] Doeringer J. A. & Hogan N. Serial processing in human movement production. Neural Networks 11, 1345–1356 (1998). 12662754 10.1016/s0893-6080(98)00083-5

[b5] Rohrer B. *et al.* Movement smoothness changes during stroke recovery. J. Neurosci. 22, 8297–8304 (2002). 12223584 10.1523/JNEUROSCI.22-18-08297.2002 PMC6758113

[b6] Lashley K. S. in Cerebral Mechanisms in Behavior ed. Jeffress L. A.) (Wiley (1951).

[b7] Gallistel C. R. The Organization of Action: A New Synthesis Erlbaum (1980).

[b8] Willingham D. B. A neuropsychological theory of motor skill learning. Psychol. Rev. 105, 558 (1998). 9697430 10.1037/0033-295x.105.3.558

[b9] Newell A. & Rosenbloom P. in Cognitive Skills and Their Acquisition (ed. Anderson J. R. 1–55Erlbaum (1981).

[b10] Heathcote A., Brown S. & Mewhort D. J. K. The power law repealed: the case for an exponential law of practice. Psychon. Bull. Rev. 7, 185–207 (2000). 10909131 10.3758/bf03212979

[b11] Miller G. A. The magical number seven, plus or minus two: some limits on our capacity for processing information. Psychol. Rev. 63, 81–87 (1956). 13310704

[b12] Verwey W. B. Concatenating familiar movement sequences: the versatile cognitive processor. Acta Psychol. 106, 69–95 (2001). 10.1016/s0001-6918(00)00027-5 11256340

[b13] Verwey W. B. & Wright D. L. Effector-independent and effector-dependent learning in the discrete sequence production task. Psychol. Res. 68, 64–70 (2004). 12955505 10.1007/s00426-003-0144-7

[b14] Verwey W. B., Abrahamse E. L. & Jiménez L. The effect of full visual feedback on the locus of an acquired nonlinear visuomotor transformation. Hum. Mov. Sci. 28, 348–361 (2009). 19135276 10.1016/j.humov.2008.10.004

[b15] Abrahamse E. L., Ruitenberg M. F. L., de Kleine E. & Verwey W. B. Control of automated behavior: insights from the Discrete Sequence Production task. Front. Hum. Neurosci. 7, 82 (2013). 23515430 10.3389/fnhum.2013.00082 PMC3601300

[b16] Shea C. H. & Kovacs A. in Individual and Team Skill Decay: The Science and Implications for Practice eds Arthur W., Day E. A., Bennett W., Portray A. M. 205–239Taylor/Francis (2013).

[b17] Hikosaka O. *et al.* Parallel neural networks for learning sequential procedures. Trends Neurosci. 22, 464–471 (1999). 10481194 10.1016/s0166-2236(99)01439-3

[b18] Hogan N. An organizing principle for a class of voluntary movements. J. Neurosci. 4, 2745–2754 (1984). 6502203 10.1523/JNEUROSCI.04-11-02745.1984 PMC6564718

[b19] Shadmehr R. & Wise S. The Computational Neurobiology of Reaching and Pointing: A Foundation for Motor Learning MIT Press (2005).

[b20] Flash T. & Hogan N. The coordination of arm movements: an experimentally confirmed mathematical model. J. Neurosci. 5, 1688–1703 (1985). 4020415 10.1523/JNEUROSCI.05-07-01688.1985 PMC6565116

[b21] Todorov E. & Jordan M. Smoothness maximization along a predefined path accurately predicts the speed profiles of complex arm movements. J. Neurophysiol. 80, 696–714 (1998). 9705462 10.1152/jn.1998.80.2.696

[b22] Graybiel A. M. The basal ganglia and chunking of action repertoires. Neurobiol. Learn. Mem. 70, 119–136 (1998). 9753592 10.1006/nlme.1998.3843

[b23] Benecke R., Rothwell J. C., Dick J. P., Day B. L. & Marsden C. D. Disturbance of sequential movements in patients with Parkinson's disease. Brain 110, 361–379 (1987). 3567527 10.1093/brain/110.2.361

[b24] Bertsekas D. P. Dynamic Programming and Optimal Control Athena Scientific (1996).

[b25] Todorov E. Efficient computation of optimal actions. Proc. Natl Acad. Sci. USA 106, 11478–11483 (2009). 19574462 10.1073/pnas.0710743106 PMC2705278

[b26] Bertsekas D. P. Approximate policy iteration: a survey and some new methods. J. Control Theor. Appl. 9, 310–335 (2011).

[b27] Miettinen K. Nonlinear Multiobjective Optimization Vol. 12, (Springer (2012).

[b28] Erez T. & Todorov E. in *IEEE/RSJ International* *Conference on Intelligent Robots and Systems (IROS)* 4914–4919 Vilamoura, Algarve, Portugal (2012).

[b29] Agostino R., Berardelli A., Formica A., Accornero N. & Manfredi M. Sequential arm movements in patients with Parkinson's disease, Huntington's disease and dystonia. Brain 115, 1481–1495 (1992). 1422799 10.1093/brain/115.5.1481

[b30] Castiello U., Stelmach G. E. & Lieberman A. N. Temporal dissociation of the prehension pattern in Parkinson's disease. Neuropsychologia 31, 395–402 (1993). 8502374 10.1016/0028-3932(93)90162-s

[b31] Phillips J. G., Chiu E., Bradshaw J. L. & Iansek R. Impaired movement sequencing in patients with Huntington's disease: a kinematic analysis. Neuropsychologia 33, 365–369 (1995). 7792003 10.1016/0028-3932(94)00114-5

[b32] Boyd L. A. *et al.* Motor sequence chunking is impaired by basal ganglia stroke. Neurobiol. Learn. Mem. 92, 35–44 (2009). 19249378 10.1016/j.nlm.2009.02.009

[b33] Izawa J., Rane T., Donchin O. & Shadmehr R. Motor adaptation as a process of reoptimization. J. Neurosci. 28, 2883–2891 (2008). 18337419 10.1523/JNEUROSCI.5359-07.2008 PMC2752329

[b34] Pekny S. E. & Shadmehr R. Optimizing effort: increased efficiency of motor memory with time away from practice. J. Neurophys. 113, 445–454 (2015). 10.1152/jn.00638.2014 PMC4297790 25355964

[b35] Franklin D. W. *et al.* CNS learns stable, accurate, and efficient movements using a simple algorithm. J. Neurosci. 28, 11165–11173 (2008). 18971459 10.1523/JNEUROSCI.3099-08.2008 PMC6671516

[b36] Barnes T. D., Kubota Y., Hu D., Jin D. Z. & Graybiel A. M. Activity of striatal neurons reflects dynamic encoding and recoding of procedural memories. Nature 437, 1158–1161 (2005). 16237445 10.1038/nature04053

[b37] Jin X. & Costa R. M. Start/stop signals emerge in nigrostriatal circuits during sequence learning. Nature 466, 457–462 (2010). 20651684 10.1038/nature09263 PMC3477867

[b38] Desmurget M. & Turner R. S. Motor sequences and the basal ganglia: kinematics, not habits. J. Neurosci. 30, 7685–7690 (2010). 20519543 10.1523/JNEUROSCI.0163-10.2010 PMC2906391

[b39] Wymbs N. F., Bassett D. S., Mucha P. J., Porter M. A. & Grafton S. T. Differential recruitment of the sensorimotor putamen and frontoparietal cortex during motor chunking in humans. Neuron 74, 936–946 (2012). 22681696 10.1016/j.neuron.2012.03.038 PMC3372854

[b40] Jin X., Tecuapetla F. & Costa R. M. Basal ganglia subcircuits distinctively encode the parsing and concatenation of action sequences. Nat. Neurosci. 17, 423–430 (2014). 24464039 10.1038/nn.3632 PMC3955116

[b41] Graybiel A. M. Vrituals, and the evaluative brain. Annu. Rev. Neurosci. 31, 359–387 (2008). 18558860 10.1146/annurev.neuro.29.051605.112851

[b42] Berkowitz A. L. & Ansari D. Generation of novel motor sequences: the neural correlates of musical improvisation. Neuroimage 41, 435–543 (2008). 10.1016/j.neuroimage.2008.02.028 18420426

[b43] Ullén F., Bengtsson S. L., Ehrsson H. H. & Forssberg H. Neural control of rhythmic sequences. Ann. NY Acad. Sci. 1060, 368–376 (2005). 16597788 10.1196/annals.1360.031

[b44] Bengtsson H. H., Ehrsson H. H., Forssberg H. & Ullén F. Dissociating brain regions controlling the temporal and ordinal structure of learned movement sequences. Eur J. Neurosci. 19, 2591–2602 (2004). 15128413 10.1111/j.0953-816X.2004.03269.x

[b45] Pessiglione M. *et al.* How the brain translates money into force: a neuroimaging study of subliminal motivation. Science 316, 904–906 (2007). 17431137 10.1126/science.1140459 PMC2631941

[b46] Mazzoni P., Hristova A. & Krakauer J. W. Why don't we move faster? Parkinson's disease, movement vigor, and implicit motivation. J. Neurosci. 27, 7105–7116 (2007). 17611263 10.1523/JNEUROSCI.0264-07.2007 PMC6794577

[b47] Turner R. S. & Desmurget M. Basal ganglia contributions to motor control: a vigorous tutor. Curr. Opin. Neurobiol. 20, 704–716 (2010). 20850966 10.1016/j.conb.2010.08.022 PMC3025075

