# Supplementary material for: Chunking as the result of an efficiency computation trade-off
Source: Nat Commun. 2016 Jul 11;7:12176. doi: 10.1038/ncomms12176 (PMC4942581; doi:10.1038/ncomms12176)
Supplement: Supplementary Information — Supplementary Figures 1 and 2 [file ncomms12176-s1.pdf]

SUPPLEMENTARY FIGURES

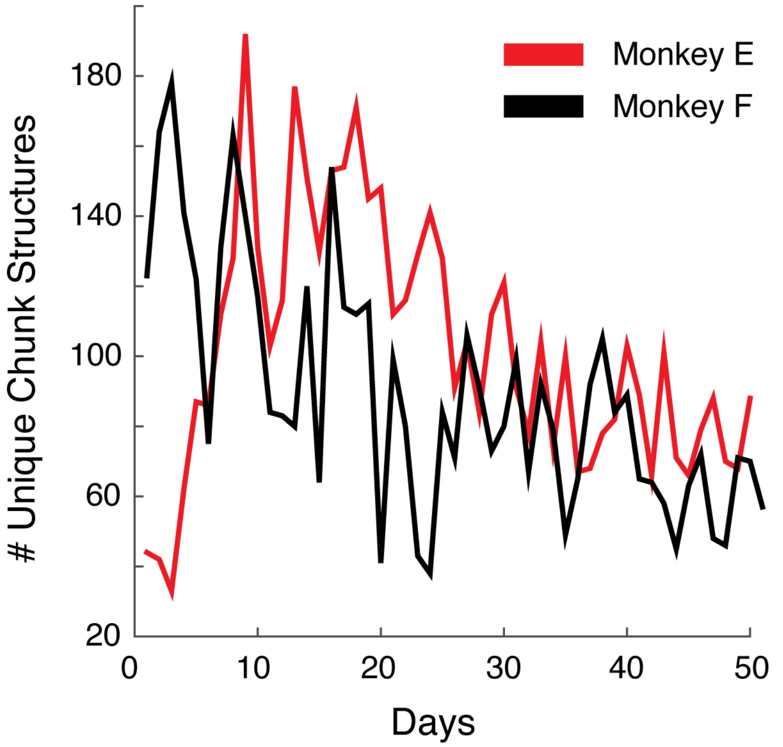

Supplementary Figure 1. Convergence of chunk structures. Monkeys start out with executing as many as 190 of 512 possible chunk structures, but converge to about 80 unique structures towards the end of learning.

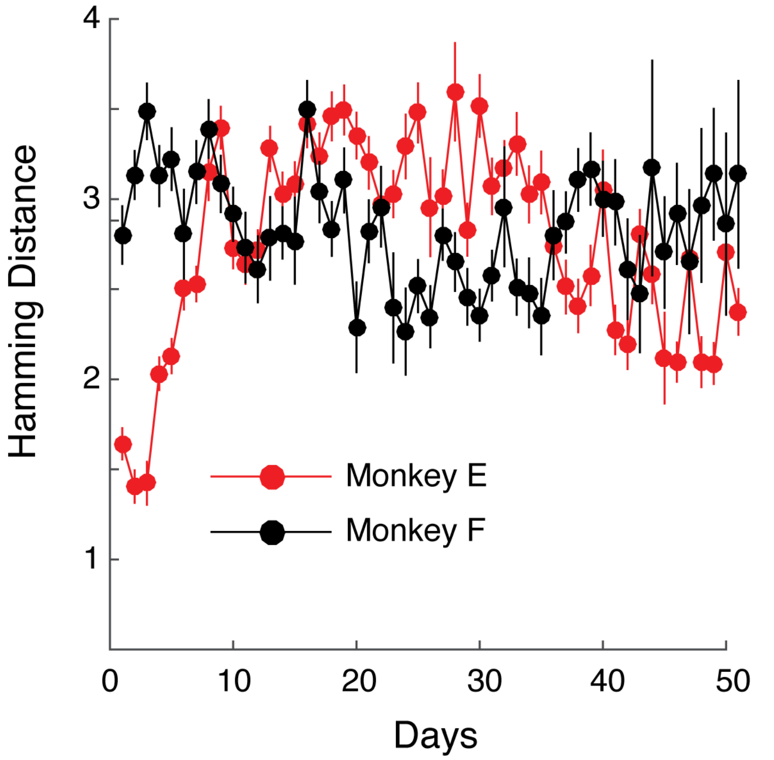

Supplementary Figure 2. Trial to trial variability of chunk structures. Hamming distance between consecutive pairs of chunk structures across sessions. Error bars show 2 SEMs.
